# Symmetric offset versus asymmetric offset ablation with transepithelial refractive keratectomy

**DOI:** 10.1186/s12886-023-02971-9

**Published:** 2023-05-17

**Authors:** Diego de Ortueta, Dennis von Rüden, Samuel Arba Mosquera

**Affiliations:** 1 Aurelios Augenlaserzentrum Recklinghausen, Erlbruch 34-36, 45657 Recklinghausen, Germany; 2 SCHWIND Eye-Tech-Solutions, 63801 Kleinostheim, Germany

**Keywords:** Transepithelial photorefractive keratectomy, TransPRK, Photorefractive keratectomy, PRK, Corneal vertex, Hyperopia, Myopia, Astigmatism, Pupil offset

## Abstract

**Background:**

In eyes with hyperopia, astigmatism, and mixed astigmatism Transepithelial photorefractive keratectomy (TransPRK) is a modality of surface ablation surgery. We center on the corneal vertex for all our treatments (all have an offset to the center of the pupil) and wanted to compare the visual results of symmetrical profile treatments versus asymmetrical profile treatments (the center of the treatment on the vertex and the boundaries with the pupil center) using TransPRK as corneal refractive surgery.

**Methods:**

We retrospectively analyzed two consecutive groups of eyes treated with TransPRK in the Aurelios Augenlaserzentrum Recklinghausen: 47 eyes treated with symmetrical offset and 51 eyes treated with asymmetrical offset. The intergroup comparisons were assessed using unpaired Student’s T-tests, whereas preoperative to postoperative changes were assessed using paired Student’s T-tests.

**Results:**

Refractive outcomes were good for both groups. 83 and 88% of eyes were within the spherical equivalent of 0.5 D from the target in the symmetric and asymmetric offset groups, respectively. 85 and 84% of eyes had a postoperative astigmatism of 0.5 D or lower in the symmetric and asymmetric offset groups, respectively.

**Conclusion:**

We have not found a significant difference in the refractive outcomes between the symmetric group and the asymmetric group of eyes treated both with TransPRK for preoperatively hyperopic or mixed astigmatism.

## Background

There is an ever-growing body of evidence that using the pupil-vertex offset (i.e. centring refractive correction on the corneal vertex) produces better outcomes. Thus, “offset”, i.e. “corneal vertex” centred refractive corrections, are an important part in the literature.

Our group has advocated centering the ablation on the vertex of the cornea for laser refractive surgery [1–4]. The corneal vertex (CV) is determined from the coaxial Coaxially sighted corneal light reflex from the first Purkinje image [5]. In our laser system, we use the information from the videokeratoscopy and displace the center of the ablation with an offset from the measured pupil center towards the vertex of the cornea; we have several publications showing good results in hyperopic LASIK centering on the vertex of the cornea [1], what other authors also describe as coaxial light [6, 7], or subject-fixed coaxially sighted corneal light reflex described by Chang and Waring [8].

At least as important as centration is to get a wide optical zone (OZ), the OZ should at least cover the pupil boundaries in mesopic conditions to avoid side effects such as glare and starburst [9]. The high order aberrations (HOA) are less with a larger OZ [10], therefore the OZ should cover the complete pupil area in the different light conditions.

There has also been an increase in the number of publications using the asymmetric offset strategy since the original publication in 2012 [11]. We also use the asymmetric offset [12, 13]. The asymmetric ablation is described in detail in a previous publication [11], but in brief, we use the CV for the centration, taking into account the boundaries of the pupil. In the last few years, we have performed more surface ablations surgeries [14–16] and used transepithelial photorefractive keratectomy (TransPRK) surgery as a standard method to correct ametropia with the excimer laser. We have been using the TransPRK with asymmetric offset centering on the CV until 2019. With TransPRK, we have the advantage that we can treat a large optical zone and are not limited by the flap diameter. The question is whether with hyperopia and mixed astigmatism treatment with symmetric offset (SO) and larger optical zones, results get better than using asymmetric offset (AO), since the treatment itself acts more symmetrically on the cornea.

To the best of our knowledge, there is not a single publication comparing the outcomes of a symmetric vs. an asymmetric offset centration strategy. Thus, a direct comparison of the outcomes of a symmetric vs. an asymmetric offset centration strategy (both placing the refractive correction on the corneal vertex) is a valid purpose for a formal evaluation.

## Methods

We analyzed retrospectively two groups of consecutive eyes with a minimum follow up of 4 months. The patients were treated by a single surgeon (DdO) in Aurelios Augenlaserzentrum Recklinghausen, Germany, between January 2017 and January 2021. The first group of consecutive eyes (47 eyes) was treated with TransPRK with SO (Group A). A previous consecutive group of 314 eyes was treated with TransPRK with AO (Group B); from this 314 we chose the last treatments with a similar number of eyes.

Patients were enrolled in the study if they had a preoperative corrected distance visual acuity (CDVA) of 0.8 or better using Snellen Charts according to the provisions set by the International Standardization Organization (ISO), stable refraction for 1 year before the study, and discontinued contact lens use for at least 2 weeks before the preoperative evaluation.

The exclusion criteria preoperative were a calculated postoperative corneal bed thickness less than 350 microns after ablation, irregular corneas or keratoconus suspected, previous ocular surgery, and diseases with ocular manifestation.

In all the cases, we performed a complete ophthalmological examination prior to surgery and after 1 and 4-months, including assessment of manifest refraction with and without correction, cyclopegic refraction, fundoscopy, and stereopsis with the Lang test. We also use the total error at 4 mm of the aberrometer Peramis (SCHWIND eye-tech-solutions, Kleinostheim, Germany) for subjective refraction and the total astigmatism from the topographer SIRIUS topo-tomographer (Costruzione Strumenti Oftalmici, Florence, Italy). The pachymetry data, topography, and pupillometry were also obtained with the SIRIUS. The best subjective refraction for distance measured by DvR with the maximum accepted sphere and less astigmatism nearest to the topographic total astigmatism was introduced as a refraction in the ORK CAM software of the SCHWIND AMARIS 1050 RS laser platform (SCHWIND eye-tech-solutions GmbH, Kleinostheim, Germany). The keratometry, pachymetry, offset (distance from the vertex of the cornea to the center of the pupil), infrared photography of the eye for static cyclotorsion was also exported from the SIRIUS topo-tomographer to the AMARIS laser system.

In all cases, the one-step TransPRK surface ablation of epithelium and stroma was used. The used ablation laser was aberration-free [17] with an aspheric profile compensating the energy loss at the periphery [18] with Smart Pulse Technology [19]. The aspheric ablation of the epithelium was standardly programmed with a value of 55 microns at the center and 65 microns at the 4 mm periphery.

The AMARIS 1050 RS laser platform uses a flying-spot delivery system that operates at 1050 Hz with a super-Gaussian beam profile of 0.54 mm full width at half maximum [20]. The laser uses a randomized flying-spot ablation pattern to minimize the thermal load of the treatment [21, 22]. A more detailed description of the laser system can be found in a previous publication [16].

After the surgery, a soft bandage contact lens (Air Optix Night & Day, base curve 8.4) was applied for 4 days. The patients took the eye drops dexamethasone Dexa edo (Dr. Mann Pharma, Bausch and Lomb, Berlin, Germany) and ofloxacin Floxal Edo eye drops (Dr. Mann Pharma, Bausch and Lomb, Berlin, Germany) four times a day for 2 weeks, fluormetholon eye drops (Fluoropos Ursapharm GmbH, Saarbrücken, Germany) three times a day for another 6 weeks, and preservative-free lubricants for 2 months as needed and beyond, if necessary.

Uncorrected distance visual acuity (UDVA) and CDVA were analyzed using the Excel software (Microsoft Corp. Redmond, Washington). The Snellen acuities were converted to logMAR for data reporting using the Visual Acuity Conversion Chart of the Journal of Cataract and Refractive Surgery. Vector analysis as described by Alpins [23] has been performed. Data for up to 4 months postoperatively are reported here.

A *P* value less than 0.05 was considered statistically significant. The normality of the samples was assessed using the back-of-the-envelope and the quantil-quantil methods. The intergroup comparisons were assessed using unpaired Student’s T-tests, whereas preoperative to postoperative changes were assessed using paired Student’s T-tests. Since the cohorts are small enough, we have applied Fisher’s exact tests to compare the proportions between groups.

There are no patients on a contralateral basis, i.e., one eye belongs to each different group. Eyes included in both groups were in general included from both patients (unless one eye did not meet the inclusion criteria). To account for that, the statistics were calculated based on the actual number of patients and not on the total number of eyes.

## Results

Three hundred sixty-one eyes met the inclusion criteria for the retrospective chart review, all with acompleted 4-month follow-up. Of those 47 eyes, corresponded to the symmetric offset (SO) cohort (study group), and the last 51 of the asymmetric offset (AO) cohort have been taken for comparison (control group). Comparative data are presented in Table 1.

**Table 1:** Demographic and refractive data of symmetric offset group and asymmetric offset group

Parameter	Symmetric Offset	Asymmetric Offset	*p*-value
Number of eyes (n)	47	51	–-
Gender (F/M) %	53%/47%	71%/29%	.04
Laterality (OD/OS) %	45%/55%	51%/49%	.3
Treatment date (days)	28/10/2020 ± 119	20/10/2019 ± 84	< .0001
Age (years)	37 ± 12	38 ± 11	.4
Optical Zone (mm)	7.1 ± 0.1	7.0 ± 0.2	.0001
Transition Zone (mm)	1.2 ± 0.3	1.3 ± 0.4	.2
Total ablation Zone (mm)	8.4 ± 0.3	8.3 ± 0.4	.2
Treatment time (s)	35 ± 8	34 ± 7	.3
Max Total ablation Depth (µm)	112 ± 23	109 ± 19	.3
Central Stromal ablation depth (µm)	23 ± 22	15 ± 19	.03
Max Stromal ablation depth (µm)	49 ± 23	45 ± 20	.2
Relative Humidity (%)	31 ± 9	35 ± 7	.02
System Temperature (°C)	26 ± 1	26 ± 1	–-
Amblyopic eyes %	0%	0%	–-
Steep keratometry (D)	44.4 ± 1.7	44.0 ± 1.8	.1
Flat keratometry (D)	42.2 ± 1.5	42.3 ± 1.6	.4
Mean keratometry (D)	43.3 ± 1.5	43.2 ± 1.6	.3
Corneal toricity (D)	2.2 ± 1.0	1.7 ± 0.8	.01
J0/90 toricity (D)	1.8 ± 1.4	0.9 ± 1.5	.002
J45/135 toricity (D)	0.0 ± 0.7	0.0 ± 0.8	.5
Central pachymetry (µm)	557 ± 37	557 ± 30	.5
Static cyclotorsion (deg)	-0.5 ± 2.3	-0.1 ± 2.7	.2
Min dynamic cyclotorsion (deg)	-0.7 ± 0.7	-1.2 ± 1.3	.01
Max dynamic cyclotorsion (deg)	0.9 ± 0.7	0.9 ± 0.9	.5
Follow-up time (months)	2 ± 1	5 ± 7	.002
Preop UDVA (logMAR)	0.4 ± 0.2	0.4 ± 0.2	.1
Postop UDVA (logMAR)	0.1 ± 0.2	0.1 ± 0.1	.3
Preop CDVA (logMAR)	0.0 ± 0.1	0.0 ± 0.1	.1
Difference UDVApost – CDVApre (Snellen lines)	-1.2 ± 1.6	-0.8 ± 1.0	.1
Postop CDVA (logMAR)	0.0 ± 0.0	0.0 ± 0.1	.1
Change in CDVA (Snellen lines)	-0.2 ± 0.6	-0.2 ± 0.8	.4
Preop manifest sphere (D)	+ 1.26 ± 0.92	+ 1.52 ± 1.17	.1
Preop manifest cylinder (D)	-2.15 ± 1.33	-1.64 ± 0.94	.02
Planned manifest sphere (D)	+ 1.27 ± 0.93	+ 1.51 ± 1.18	.1
Planned manifest cylinder (D)	-2.15 ± 1.33	-1.64 ± 0.95	.02
Postop manifest sphere (D)	+ 0.09 ± 0.37	+ 0.11 ± 0.35	.4
Postop manifest cylinder (D)	-0.31 ± 0.29	-0.33 ± 0.35	.4

**Fig. 1:**
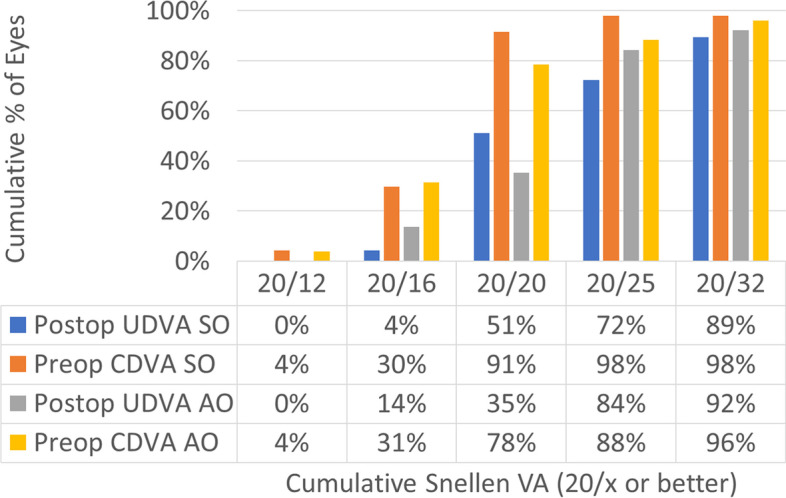
Graphic bar showing the cumulative Snellen acuity graphic for comparison of preoperatively corrected distance visual acuity (CDVA) and 4 month postoperatively uncorrected distance visual acuity (UDVA) for both groups Simmetrical Offset (SO) and Asymmetrical Offset (AO)

**Fig. 2:**
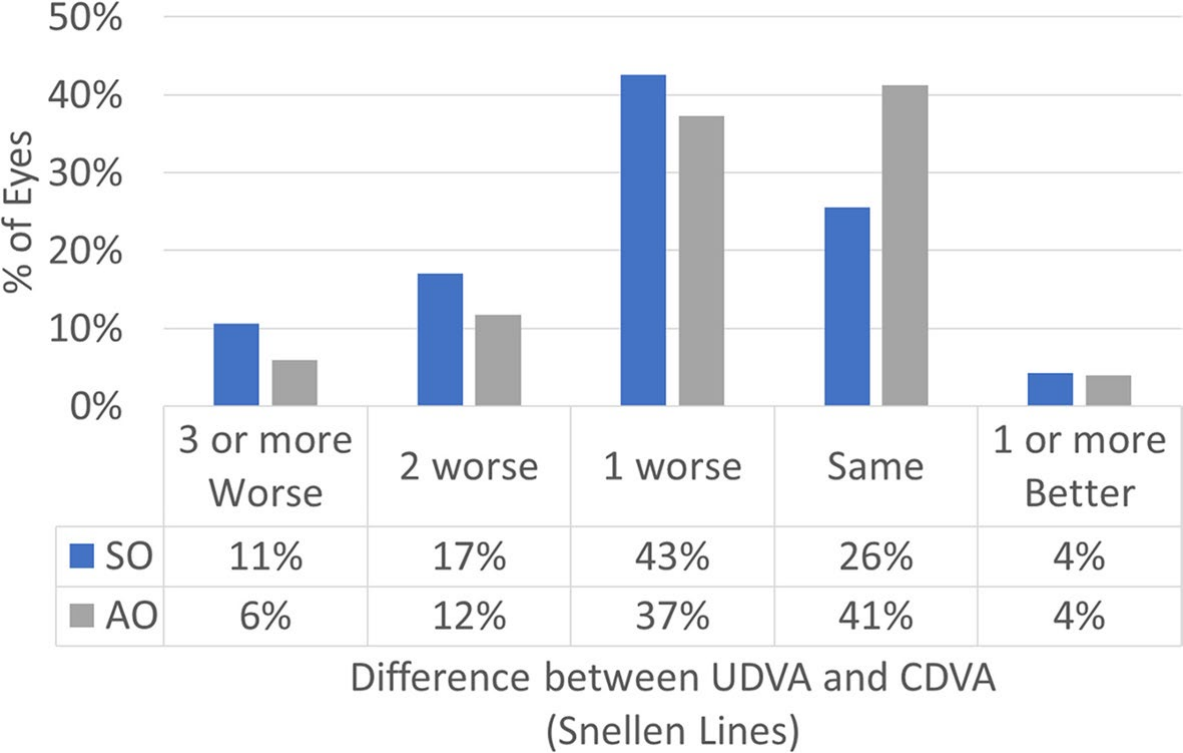
Efficacy of both groups, AO versus SO Showing in bars the gain or loss of Snellen lines comparing preoperative CDVA with postoperative UCDVA after 4 months of TransPRK for both groups, SO in blue and AO in grey

**Fig. 3:**
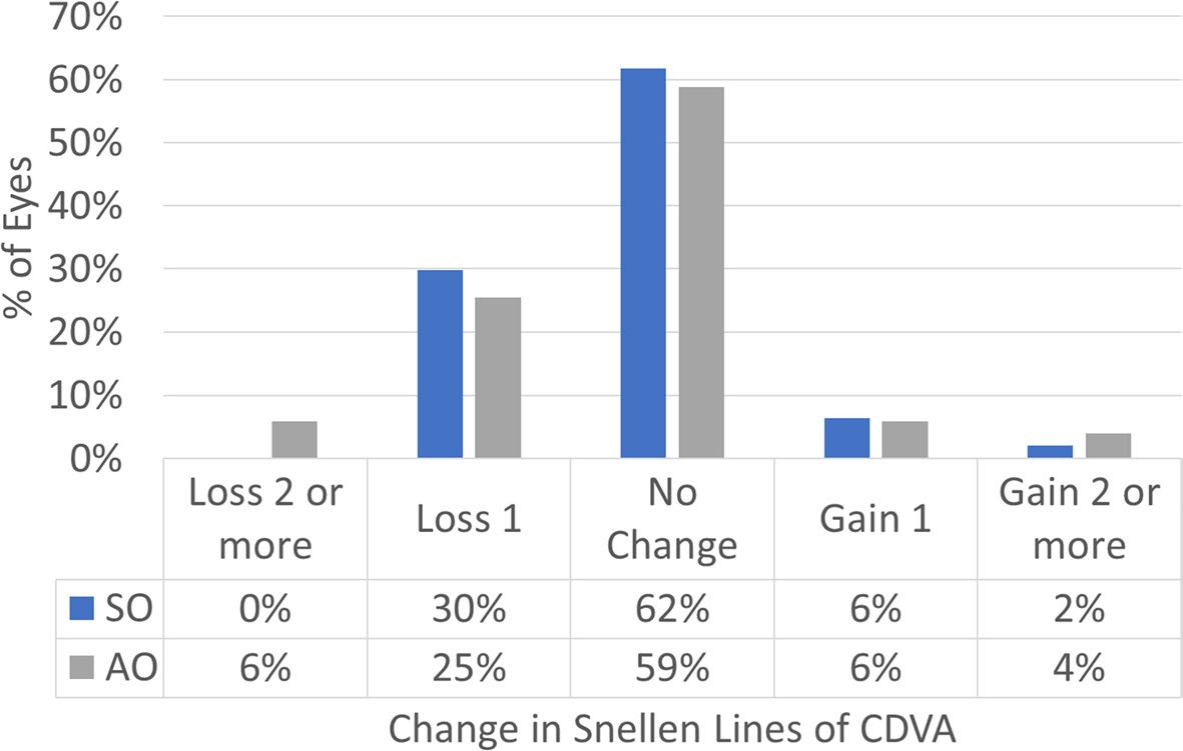
Gain and loss of lines of best CDVA 4 months postoperatively after TransPRK in the groups SO in blue and AO in grey

**Fig. 4:**
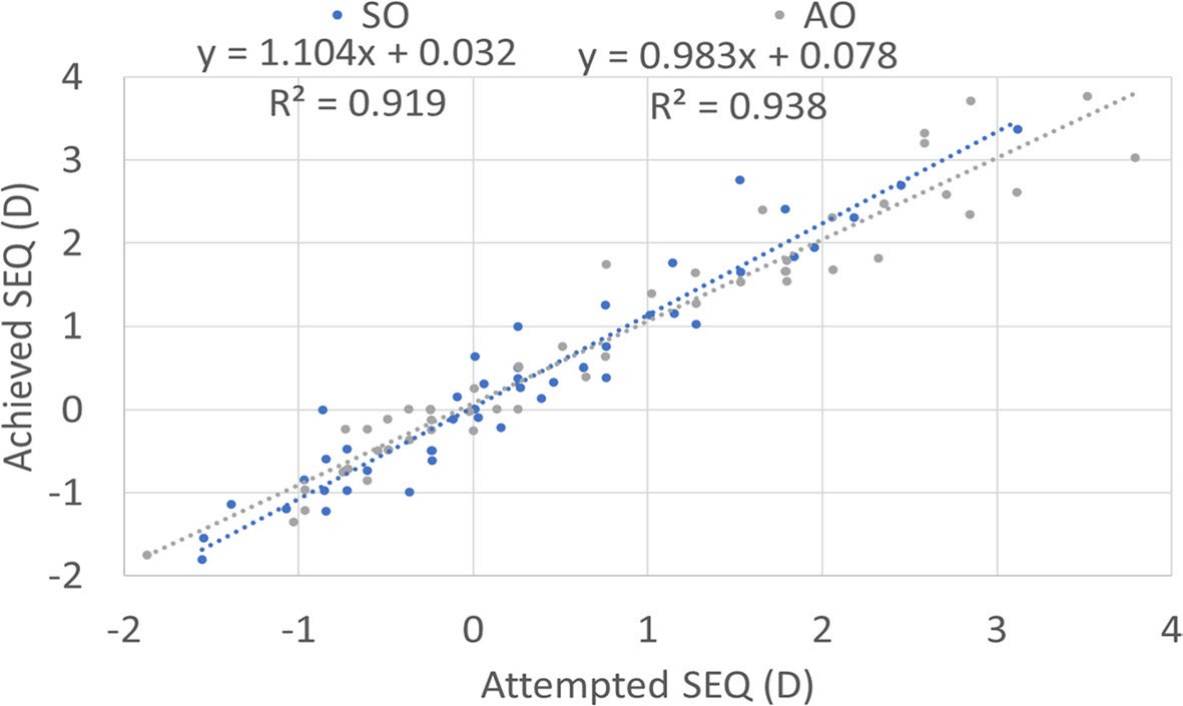
Achieved spherical equivalent (SEQ) versus attempted SEQ after 4 months of TransPRK. Both groupos SO and AO have coefficients of determination R2 near 1

**Fig. 5:**
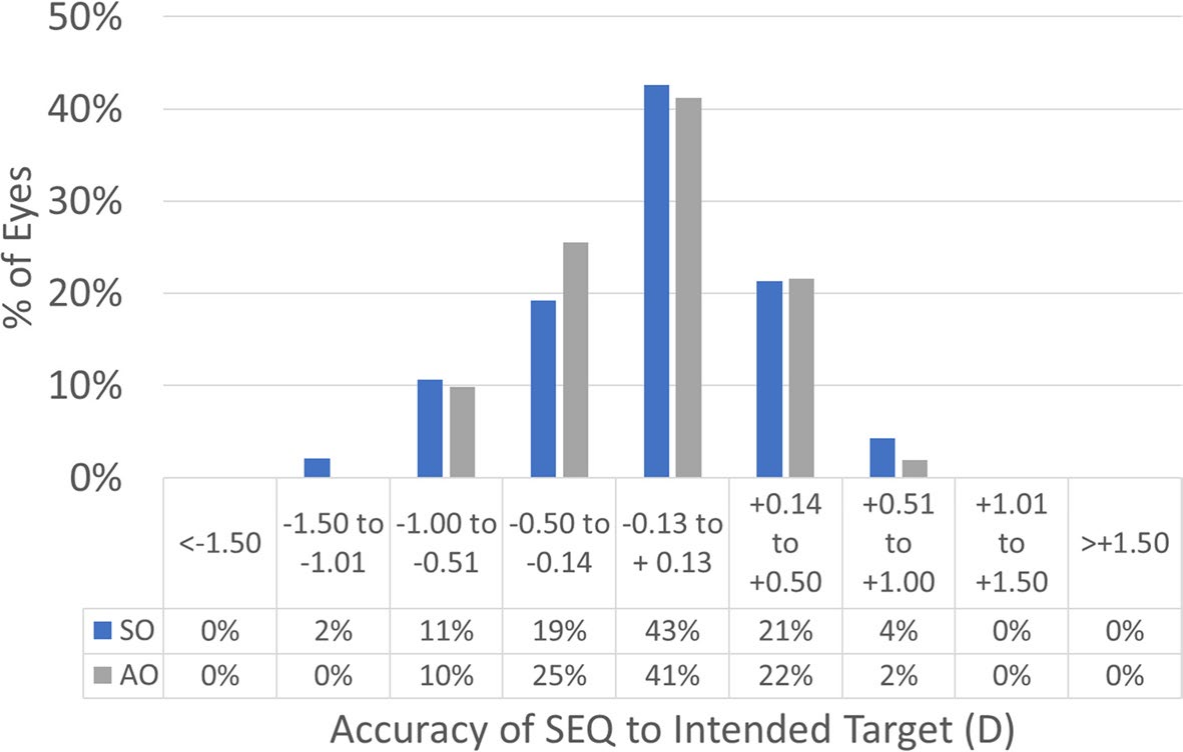
Accuracy of the TransPRK treatment with SO and AO Bars showing the intended target and the achieved SEQ after 4 months of TransPRK

**Fig. 6:**
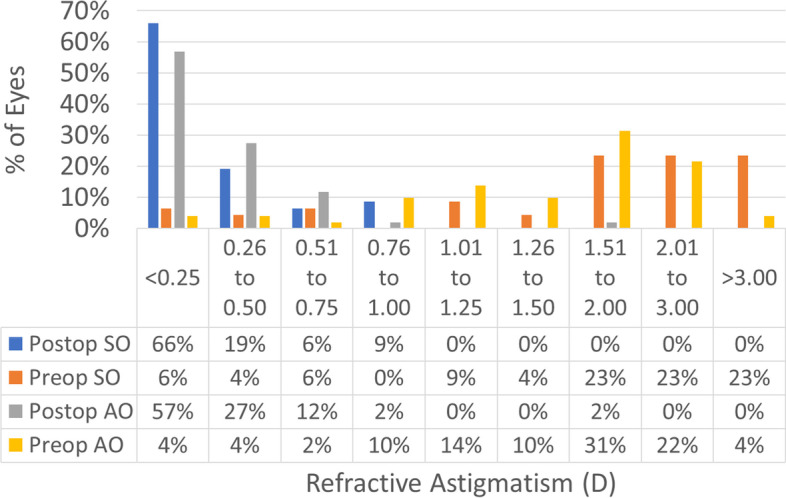
Analysis of the Astigmatism Preoperative astigmatism and postoperative astigmatism after 4 months TransPRK

**Fig. 7:**
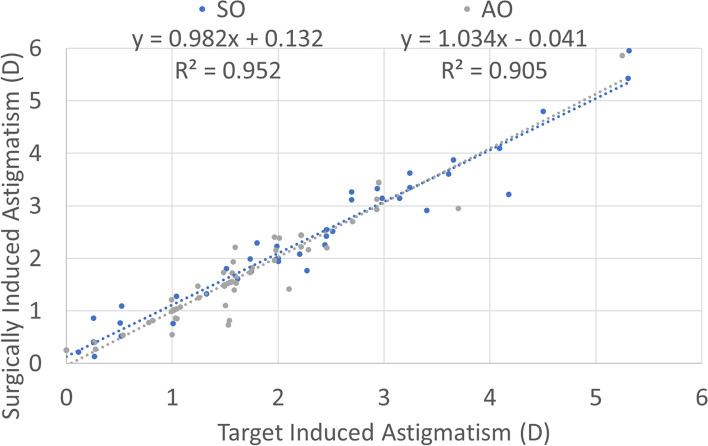
Induced astigmatism after 4 months of trans-PRK. A histogram comparing target and surgically induced astigmatism.The coefficient of determination is near 1, showing good correction of the astigmatism in both groups SO and AO

**Fig. 8:**
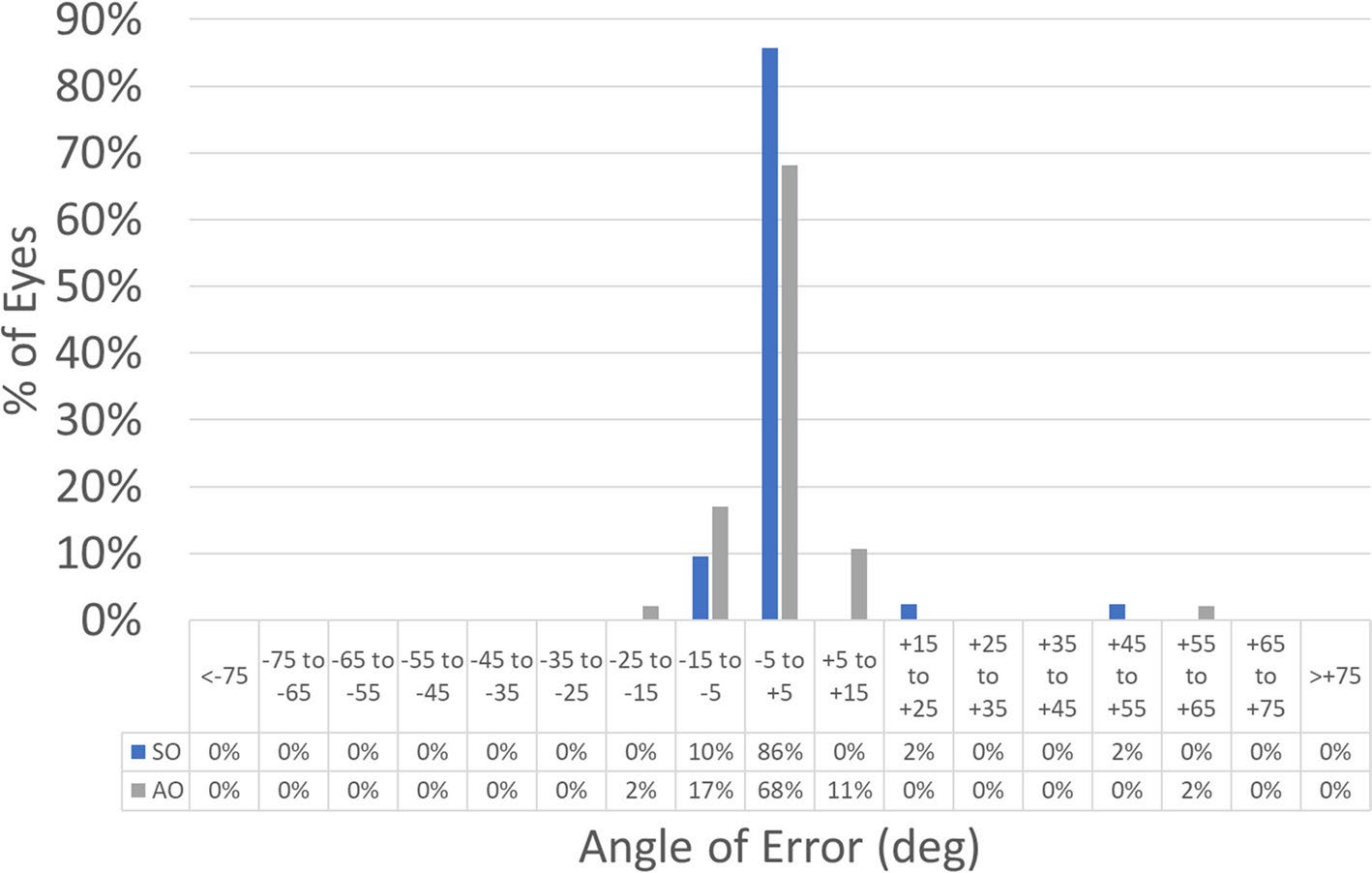
Angle of error of the correction of astigmatism after 4 months of TransPRK with SO and AO Most of the cases were near 5 degrees for both groups

Treatments in the SO group had a 0.1 mm larger OZ than treatments in the AO group (7.1 ± 0.1 mm vs. 7.0 ± 0.1 mm; *p*-value = 0.0001) this also implicates that the SO group had a deeper central ablation than treatments in the AO group (23 ± 3 µm vs. 15 ± 3 µm; *p*-value 0.02). The refractive results are presented in Figs. 1, 2, 3, 4, 5, 6, 7 and 8.

Figure 1 shows the cumulative Snellen acuity, with 72 and 84% of eyes achieving a postoperative UDVA of 20/25 or better for the SO and AO groups, respectively. Figure 2 displays the efficacy of both groups, with 73 and 82% of eyes achieving a postoperative UDVA within 1 line from the preoperative CDVA for the SO and AO groups, respectively. Figure 3 depicts the change in CDVA 4 months postoperatively after TransPRK, with no eye losing 2 or more lines of CDVA in the SO group and 6% of eyes losing 2 or more lines of CDVA in the AO group. Figure 4 shows a scattergram of achieved vs. attempted spherical equivalent; the SO group shows a moderate overcorrection (11%) vs. a slight undercorrection (-1%) for the AO group. Figure 5 shows the accuracy of the TransPRK treatment in terms of SEq, with 83 and 88% of eyes within 0.5D of the target defocus for the SO and AO groups, respectively. Figure 6 shows the accuracy of the TransPRK treatment in terms of refractive astigmatism, with 85 and 84% of eyes within 0.5D of astigmatism for the SO and AO groups, respectively. Figure 7 shows a scattergram of achieved vs. attempted cylinder correction; both groups show excellent and accurate correction. Figure 8 shows the accuracy of the TransPRK treatment in terms of astigmatism axis, with 96% of eyes within 15 degrees of the attempted astigmatic axis for either group.

In terms of refractive outcome Preoperative SEq in the SO group was lower than in the AO group (0.2 ± 0.2D vs. 0.7 ± 0.2D; *p*-value 0.02). In terms of astigmatism, the preoperative astigmatism was higher in the SO group than in the AO group (2.2 ± 0.2D vs 1.6 ± 0.1D; *p*-value 0.02). The preoperative with-the-rule refractive astigmatism in the SO group was higher than in the AO group (0.7 ± 0.1D vs. 0.2 ± 0.1D; *p*-value 0.003), but postoperatively there was no difference between groups (*p*-value 0.4). Refractive outcomes were unremarkable for both groups. 72 and 84% of eyes reached UDVAs of 20/25 or better in the symmetric and asymmetric offset groups, respectively. 29 and 45% of eyes achieved a postoperative UDVA as good as or better than the preoperative CDVA in the symmetric and asymmetric offset groups, respectively. 8 and 10% of the eyes gained lines of CDVA in the symmetric and asymmetric offset groups, respectively. 83 and 88% of eyes were within 0.5D of the target in the symmetric and asymmetric offset groups, respectively. 85% and 84% eyes had a postoperative astigmatism of 0.5D or lower in the symmetric and asymmetric offset groups, respectively. In either group, 96% of the eyes were within 15 degrees of the planned astigmatism axis.

The absolute angle of error, defined as the angular displacement between the surgically induced and the target-induced astigmatism,was lower in the SO group than in the AO group (3 ± 1 deg vs 6 ± 1 deg; *p*-value of 0.04). The correction index was higher in the SO group than in the AO group (1.1 ± 0.1 vs 1.0 ± 0.1; *p*-value of 0.03), and the magnitude of error defined as difference between the magnitudes of surgically induced and target-induced astigmatism in the SO group was lower than in the AO group (0.2 ± 0.1D vs 0.4 ± 0.1D; *p*-value 0.0002).

If we compare with the keratometric data, the steepening/flattening effect in the SO group was higher than in the AO group (2.4 ± 0.2D vs. 1.7 ± 0.1D; *p*-value of 0.003). The flattening index, defined as the ratio of flattening effect to target induced astigmatism, was higher in the SO group than in the AO group (1.0 ± 0.1 vs. 0.9 ± 0.1; *p*-value of 0.04). And the coefficient of adjustment, defined as the reciprocal of the correction index, in the SO group was lower than in the AO group (1.0 ± 0.1 vs. 1.3 ± 0.1; *p*-value of 0.002).

We have not found any statistically significant or clinically relevant difference in the postoperative outcomes when comparing both centration groups. After adjusting the analysis for covariates and confounding factors, the mean values (and their differences) do not vary much, and the p-values become larger (being farther apart from statistical significance), so that the absence of statistically significant or clinically relevant differences is confirmed.

## Discussion

We know that in refractive surgery we have the possibility to center on the pupil or in the vertex of the cornea [5]. We advocate for centering on the vertex of the cornea, as we have a stable morphologic reference that is reproducible with videokeratoscopy or tomography [1–3], the center of the pupil moves depending on light conditions and is therefore not so reproducible [2]. Many groups advocate also for centering on the vertex of the cornea [6, 24], and Waring and Chan describe in a theoretical paper also the difficulties with the nomenclature of this point [7]. Symmetric ablation can be centered on the pupil or on the vertex. In the case of symmetric ablation centered on the vertex, we have a displacement of the ablation from the center of the pupil to the vertex of the cornea. In contrast, the asymmetric offset uses the vertex as the center of the ablation and the pupil as the center of the optical zone, as the treatment is concentric to the pupil center. Arba-Mosquera and Ewering described in a theoretical paper the centration strategy combining the pupil and the corneal vertex [11]. If we want to center on the vertex of the cornea, one of the advantages of the asymmetric offset is that the required ablation depth diminishes as the center is the vertex and the boundaries are the pupil theoretically, a smaller optical zone covers the pupil boundaries. The ablation profiles are such that the edges of the optical zone are concentric to the pupil cente with the optical axis coincident with the corneal vertex [11]. Mathematically, the asymmetric ablation is identical to the symmetric offset; after removing the tilt component, the ablation minimizes the depth, and the diameter is smaller, reducing the amount of tissue removed by the excimer laser.

We have published good results for hyperopic LASIK using an asymmetric offset [12]. The advantage of the asymmetrical versus the symmetrical offset is that, using the same OZ, the functional optical zone [25] is bigger with the asymmetrical profile. In LASIK, our Total Ablation Zone (Optical Zone + Transition zone) cannot be bigger than the exposed stroma after preparing the flap, taking into account that the ablation is normally moved nasally as the hyperopic eyes have the vertex in a nasal position [26]. With the TransPRK technique, we have a surface ablation, and therefore, the residual stroma is thicker than the intrastromal techniques such as LASIK or SMILE. We can also use a larger optical zone and transition zone as we are not limited by the flap diameter and are less limited by the stroma thickness, as normally we leave at least more than 300 microns of stroma after the ablation. In the case of surface ablation with TransPRK, the symmetrical ablation is at least as good as the asymmetrical treatment, first because the ablation is more uniform, as the name indicates, and produces more symmetrical changes in the whole cornea. The symmetric group had a significantly larger OZ, and the central ablation was also deeper. We used on both profiles (AO and SO) large optical zones to minimize the effect of aberrations [10]. The difference in both groups is not clinically significant enough to show differences in the UDVA.

Since there are no previous reports directly comparing symmetric with asymmetric offset from a clinical perspective (but only the seminal work from a theoretical perspective [11]). We remark and emphasize the comparison between two alternative corneal vertex (or visual axis) centrations, but we are not elucidating whether corneal vertex (or visual axis) centration is superior to pupil center centration (which is the topic of previous publications [1, 12].

A general discussion on the adequate approach to center corneal refractive surgery can also be found in the literature [27], whereas the impact of “missing the target” of induced decentrations is also formally described [28, 29]. At the end of the day, decentration may occur from a “wrong design” of the ablation strategy (i.e., planning the treatment for a sub-optimal reference) or may also be the result of eye drifts (unlike stochastic eye movements) during treatment [30].

The dilemma about proper centration is not native or unique to ablation procedures, but to any refractive procedure, or in our case, all forms of corneal refractive surgery. Lenticule extraction is one alternative approach, which has also been evaluated for centration [24, 31, 32]. As presented in the table, several parameters were different between the SO and AO groups at the preoperative baseline. These may act as confounding factors. We have honestly disclosed all detected and measured parameters for both groups in the table, detailed enough to provide this differential baseline. We have preferred not to omit this information and have noticed the differences. With the relative small size of both cohorts, it would be statistically difficult to account for confounding factors. Further limitations include the retrospective nature of the comparison, a minimum follow-up of 4 months, a relatively small sample size, or bilaterally treated patients.

In this paper, we have used a larger optical zone with a symmetric offset to cover the pupil as we need to cover the pupil boundaries, and therefore, we have used a deeper ablation. In terms of efficiency and safety, both asymmetric and symmetric with offset designs were similar. The astigmatism was treated similarly, with a symmetric or asymmetric ablation profile centering on the vertex. Future studies about remodeling after TransPRK with asymmetric or symmetric profiles will show if there are differences in the epithelium.

## Conclusions

With symmetric ablation centering on the vertex of the cornea (CV), we have not found a significant difference in the achieved astigmatism between the symmetric group and the asymmetric group of eyes treated both with TransPRK with preoperatively hyperopic or mixed astigmatism.

## Data Availability

Data are available under request.
